# Expression of Transglutaminase in Foreskin of Children with Balanitis Xerotica Obliterans

**DOI:** 10.3390/ijms17091551

**Published:** 2016-09-14

**Authors:** Tiziana Russo, Monica Currò, Anna Barbera, Daniela Caccamo, Pietro Antonuccio, Salvatore Arena, Angela Simona Montalto, Saveria Parisi, Lucia Marseglia, Eloisa Gitto, Riccardo Ientile, Pietro Impellizzeri, Carmelo Romeo

**Affiliations:** 1 Department of Human Pathology of Adult and Childhood “Gaetano Barresi”, University of Messina, Messina 98125, Italy; russotiziana82@gmail.com (T.R.); pantonuccio@unime.it (P.A.); salvatore.arena@gmail.com (S.A.); asmontalto@libero.it (A.S.M.); parisi.saveria@libero.it (S.P.); lmarseglia@unime.it (L.M.); egitto@unime.it (E.G.); romeoc@unime.it (C.R.); 2 Department of Biomedical and Dental Sciences and Morphofunctional Imaging, University of Messina, Messina 98125, Italy; moncurro@unime.it (M.C.); anna.barbera1@virgilio.it (A.B.); dcaccamo@unime.it (D.C.); ientile@unime.it (R.I.)

**Keywords:** balanitis xerotica obliterans, phimosis, foreskin, transglutaminase, IFN-γ, E-cadherin

## Abstract

Balanitis xerotica obliterans (BXO) is a chronic inflammatory skin disorder of unclear etiology. The etiology and the exact molecular mechanisms underlying the disease are still unknown. The human transglutaminase (TG) family consists of several proteins with catalytic activity essential for biological processes. In the present research we investigated the transcript levels of three TGs in patients operated on for congenital phimosis without or with histologically confirmed BXO; Thirty children with acquired phimosis were enrolled. The removed foreskins were sent both for histological diagnosis and for quantitative real-time PCR to evaluate the transcript levels of keratinocyte (TG1), tissue (TG2), and epidermal (TG3) transglutaminase; We observed a decrease in TG1 and TG3 transcripts by about 70% (*p* < 0.001) in foreskins from patients with BXO (*n* = 15) in comparison with patients without BXO (*n* = 15) and an increase in TG2 mRNA levels by 2.9 folds (*p* < 0.001); Reduced expression of both TG1 and TG3 was associated with the altered structure of the foreskin in BXO and can be a consequence of damage to keratinocytes. Increased expression of TG2 can be the result of chronic inflammation. TG2 overexpression can play a pivotal role in triggering and maintaining the inflammatory response in BXO patients.

## 1. Introduction

Balanitis xerotica obliterans (BXO) is a chronic progressive inflammatory condition of unclear etiology and is considered the male genital variant of lichen sclerosus (LS). Phimosis is the most common presenting symptom. BXO was first reported by Stuhmer in 1928 and initially described in the literature as a “post-circumcision phenomenon” [1].

BXO most commonly presents in the genital region as white atrophic skin lesions of the glans frenulum, meatus and urethra. However, BXO may be complicated by meatal and urethral stenosis and maybe associated with a higher incidence of high-risk penile carcinoma [2]. The etiology of this disease still remains unknown, but it has been reported to have a frequent association with autoimmune diseases, thyroid disease, alopecia areata, vitiligo, and pernicious anemia [3,4].

Although BXO can be detected clinically, the diagnosis is based on histopathological examination. The histopathology of BXO is characterized by alterations in the epidermis and dermis as well as inflammatory cell infiltration. The epidermis is usually atrophic and often with a homogeneous zone with vacuolar degeneration of the basal layer. The superficial dermis is edematous and hyalinized [5]. In the advanced stages, dermal collagen forms a homogenous band at the dermal-epithelial junction in conjunction with elastin fibers to produce an amorphous hybrid substance. Deep to this amorphous band, a chronic inflammatory cell infiltration, mainly from T cells, is present [5]. Inflammation involves all skin layers; in particular, in the papillary dermis the inflammatory process produces alterations in fibroblast function, leading to fibrosis of the upper dermis. In this regard, several factors may be involved in the underlying molecular changes associated with the inflammatory response, including metalloproteinases, growth factors, cytokines and other enzymes [6].

Transglutaminases (TGs) are a family of structurally and functionally related enzymes which catalyze the Ca^2+^-dependent posttranslational modification of proteins. The most widely distributed and most abundantly expressed member is tissue type TG (TG2) that, in addition to transamidation activity, has other enzymatic and non-enzymatic activities. For instance, TG2 can bind and hydrolyze GTP [7], exhibits protein disulphide isomerase activity [8], acts as a protein kinase independently of calcium [9], and interacts with extracellular proteins [10]. Given its several biochemical activities, TG2 is essential for different biological processes such as cell adhesion [11], growth, migration, differentiation, programmed cell death, extracellular matrix (ECM) assembly [12,13] and wound healing [14], but can also contribute to the pathophysiology of various inflammatory, autoimmune and degenerative conditions [15,16]. 

Two other members of the TG family, TG1 and TG3, are predominantly expressed in the keratinizing epithelia, such as the epidermis, that produces a toughened structure, the cornified cell envelope, which is a major component of the epithelial barrier at the tissue surface [17]. TG1 is a membrane-bound TG isozyme abundantly expressed in the upper spinous and granular layers of the skin. TG1 is associated mainly with proliferation and terminal differentiation of keratinocytes [18] and plays an essential role in cell envelope formation by the cross-linking of precursor proteins, such as loricrin and involucrin [19,20]. Also TG3 is involved in epidermal keratinization and cornified envelope formation [21]. Indeed, TG3 is a soluble enzyme expressed in differentiating keratinocytes and corneocytes [22], but it is also found in extracellular compartments [23]. Several findings indicate that both TG2 and TG1 are present in different stratified squamous epithelial and non-epithelial cells depending on tissues and their properties [21]. In line with these observations, the aim of this study was to investigate transcript levels of different TGs together with interferon-gamma (IFN-γ) and E-cadherin in the foreskin of children operated on for congenital phimosis without or with histologically confirmed BXO, to characterize the relationship between changes in TG levels and BXO.

## 2. Results

**Figure 1:**
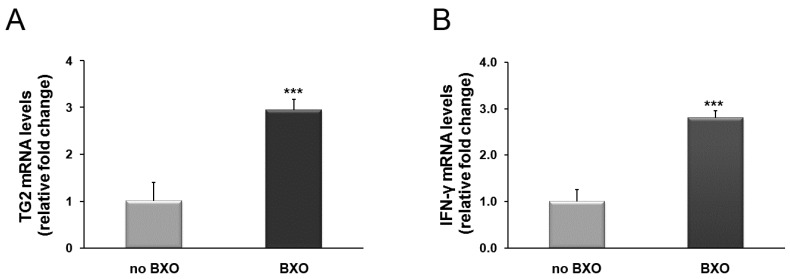
Changes in mRNA levels of TG2 (**A**) and IFN-γ (**B**) in foreskin tissues from patients with BXO expressed as relative fold changes compared with patients without BXO (no BXO). The results are the means of data obtained from 15 patients with BXO and 15 patients without BXO. Error bars represent standard error of the mean (SEM). *** *p* < 0.0001 shows significant differences in comparison with patients without BXO.

Foreskin samples from patients with and without BXO were examined for the TG2 and IFN-γ mRNA levels. We found a significant increase in the expression of TG2 and IFN-γ transcripts by 2.9- and 2.8-fold, respectively (*p* < 0.001), in foreskin from BXO patients in comparison to patients without BXO (Figure 1A,B).

**Figure 2:**
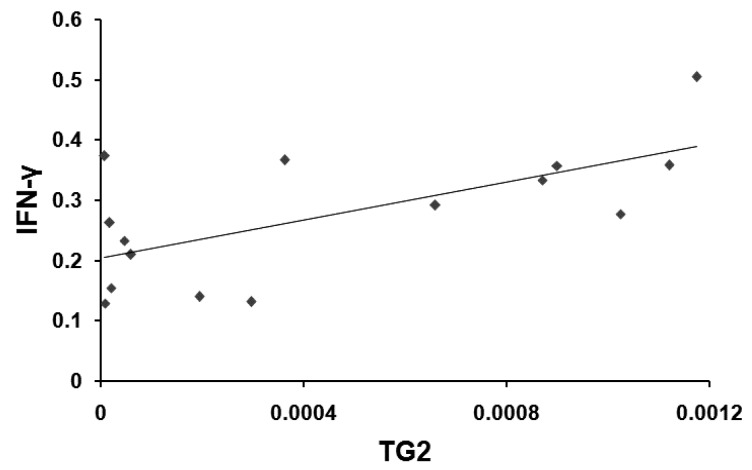
Relationship between TG2 and IFN-γ mRNA levels in foreskin from patients with BXO. TG2 mRNA transcript amount showed a positive correlation with the IFN-γ mRNA (*r* = 0.653; *p* = 0.008).

In order to emphasize the involvement of TG2 expression in the inflammatory response, we evaluated a possible correlation between TG2 and IFN-γ mRNA levels. Indeed, TG2 and IFN-γ transcripts in foreskin tissues from BXO patients were significantly and positively correlated (*r* = 0.653, *p* = 0.008) (Figure 2).

**Figure 3:**
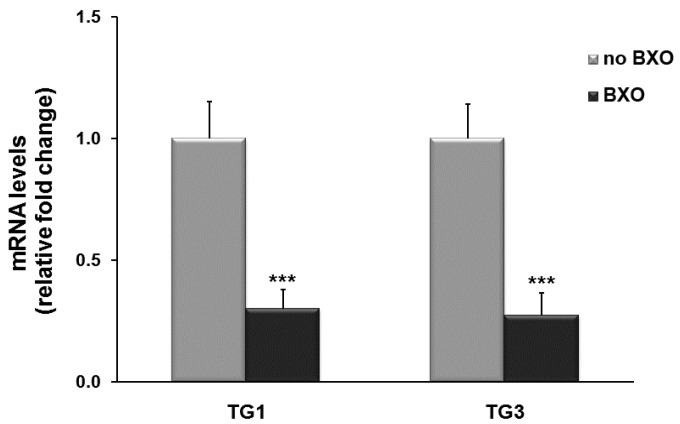
TG1 and TG3 transcript levels in foreskin tissues from patients with and without BXO (no BXO). Results from real-time PCR are expressed as relative fold change compared with foreskin from patients without BXO (no BXO). The data are the means ± standard error of the mean (SEM). *** *p* < 0.0001 shows significant differences in comparison with patients without BXO.

The expression of TG1 and TG3 mRNA was also examined. TG1 and TG3 mRNA transcripts were less abundant in tissues from BXO patients than in those from other patients. In particular, mRNA levels of both genes significantly decreased by about 70% (*p* < 0.001) in foreskin from BXO patients compared to patients without BXO (Figure 3).

**Figure 4:**
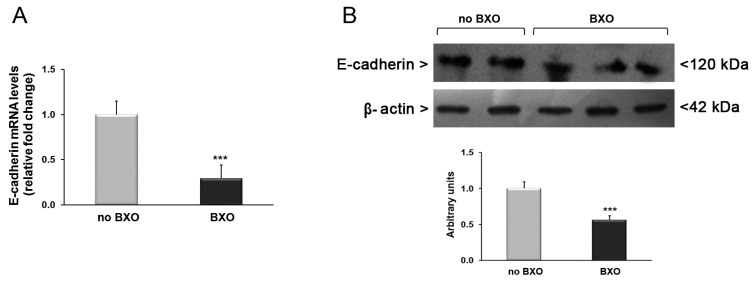
Analysis of expression levels of E-cadherin in foreskin tissues from patients with (*n* = 15) and without (*n* = 15) BXO. (**A**) Results obtained by real-time PCR are expressed as relative fold change compared with patients without BXO. Error bars represent standard error of the mean; (**B**) Western blot analysis of E-cadherin protein amounts. This picture is representative of foreskin tissues from patients with and without BXO. A representative densitometric analysis of all samples is also reported (**bottom**). The results are expressed as mean ± SEM. *** *p* < 0.0001 significant differences in comparison with patients without BXO.

Finally, we examined the expression of E-cadherin mRNA and protein amounts. The analysis by real-time PCR revealed a significant decrease in E-cadherin mRNA levels in tissues from BXO patients by 70% compared to patients without BXO (*p* < 0.001) (Figure 4A). This result was confirmed by Western blot analysis showing a reduction of E-cadherin protein expression by about 50% in foreskin samples obtained from patients with BXO in comparison to patients without BXO (Figure 4B).

## 3. Discussion

BXO is a lymphocyte-mediated chronic inflammatory disease and is one of the potential causes of acquired phimosis in childhood [24].

BXO is well characterized histologically, showing a zone of inflammatory cells with hydropic degeneration of the basal keratinocytes, and also exhibiting fibrotic and neoplastic features. 

The true incidence of LS underlying the BXO in children is underestimated [25,26]. However, the molecular mechanisms underlying the pathogenesis of BXO in children have been poorly explored until now, while several studies focused on the alterations of molecular pathways in foreskin or especially vulva tissues of adults affected by LS [27,28].

It is debated if adult and pediatric diseases represent the same disease entity. Recently, microarray technology was employed to compare the gene expression profiles of healthy and diseased prepuces obtained at circumcision in both adult and pediatric males [29]. The authors showed concordance of expression profiles between adult and pediatric samples, indicating the same disease process. In particular, it has been demonstrated that most of the genes that showed increased expression in both adult and pediatric LS were associated with immune response, including antigen processing, lymphocyte activation, chemokine activity, regulation of T cell activation and apoptosis. However, the overall trend of gene expression proved a non-specific inflammatory response, strengthening the hypothesis that male lichen sclerosus of foreskin is a chronic non-specific inflammatory disease [29]. 

Recently, molecular differences in foreskin from boys with and without lichen sclerosus with absolute phimosis have been investigated [30]. Lichen sclerosus was characterized by the overexpression of several genes associated with tissue remodeling and inflammation, including matrix metalloproteinases 1 and 9, tissue inhibitor of metalloproteinases 1, cytokine chemokine ligands 5 (RANTES), interleukin 4, transforming growth factor-β2 and its corresponding receptor [30].

Other studies evidenced an inflammatory response in genital LS; the expression of several pro-inflammatory cytokines, such as IFN-γ, TNF-α, IL-1α and IFN-γ receptor, was increased in specimens of vulval lichen sclerosus from adult women compared with specimens of normal vulva and normal skin [6,31].

Previously, cytokines such as IFN-γ and IL-6 have been studied in specimens of lichen planus, indicating that these proinflammatory cytokines are produced not only by activated T lymphocytes, but also by altered keratinocytes. Therefore, it has been hypothesized that stimulated keratinocytes may amplify the clinical course of lichen planus, promoting the infiltration of the epidermis by lymphocytes [32]. 

In this regard, we observed an increase in IFN-γ mRNA levels in foreskin obtained from children with BXO in comparison to patients without BXO. Thus, as previously demonstrated in lichen planus [32], we can hypothesize that the up-regulation of IFN-γ detected in BXO may play an important role in the induction of lymphocyte-dependent damage of keratinocytes contributing to the maintenance and progression of BXO disease.

Much evidence emphasizes the role of TG2 in inflammatory states. Indeed, cytokines and growth factors secreted during early phases of cell injury induce the increase in TG2 expression [33]. In a model of celiac sprue it has been also demonstrated that IFN-γ is able to induce the expression and the activity of TG2 [34]. Therefore, the increase of IFN-γ reported in our study may be related to the induction of TG2 observed in the foreskin of BXO patients. In addition, the positive correlation between TG2 and IFN-γ mRNA found in patients with BXO suggests that both proteins may play a role in inducing and maintaining the inflammatory response in the foreskin affected by BXO. In this regard, other findings sustain the hypothesis that the up-regulation of TG2 can contribute to the development of chronic inflammation by stimulating NF-κB activity [15]. The NF-κB transcription factor has an essential role in inflammation, largely due to its role in the induction of pro-inflammatory genes, including cytokines, chemokines, and adhesion molecules [35]. In the last years, several reports showed the existence of a TG2/NF-κB positive feedback loop, in which NF-κB directly up-regulates the transcription of TG2 and, in turn, TG2 activates NF-κB via a non-canonical pathway [36,37,38,39], leading to a persistent activation of the NF-κB pathway.

The prolonged up-regulation of TG2 in foreskin of BXO patients could be also implicated in fibrosis and the scarring process. Abnormalities in the extracellular matrix (ECM) turnover are an important factor in cutaneous fibrogenesis. Extracellular activation of TG2 contributes to the stabilization of the ECM and promotes cell-substrate interaction and wound healing [40]. Much evidence indicates that TG2 may be involved in the scarring of the lung, liver, and heart [41,42,43]. In addition, in experimental renal scarring TG2 has been associated with the accumulation of ECM components, both directly by the cross-linking of ECM proteins and indirectly via TGF-β1 activation [44].

Also, IFN-γ may contribute to the scarring and fibrosis observed in BXO, although a controversial role in fibrosis, with numerous studies showing either pro-fibrotic [45,46,47] or anti-fibrotic effects, has been reported [48,49,50].

We demonstrated that TG1 and TG3 are also expressed in foreskin specimens from patients with or without BXO. The products of their enzyme activity are associated with different biological processes depending on the location of the target proteins. The intracellular TG1 and the soluble TG3 act cooperatively in the formation of the epidermis by cross-linking of different structural proteins in keratinocytes [51,52,53]. TG1 is abundantly expressed in differentiating stratified squamous epithelia, and is essential for facilitating epidermal regeneration [54]. TG3 is highly expressed in the late differentiating keratinocytes and corneocytes [23], contributing to the epidermal barrier function. Using TG3-knockout mice, it has been demonstrated that TG3 deficiency is associated with a reduced inflammatory threshold and latent barrier defects [55]. Previously, we showed a decrease in TG1 and TG3 expression in gingival tissues affected by periodontal disease, indicating that the expression of these enzymes may be modified in response to chronic inflammation in the damaged tissue [56]. Similarly, in the present study both TG1 and TG3 were significantly down-regulated in tissues from patients with BXO compared to patients without the disease. Thus, the here-reported results suggest that the observed changes in TG1 and TG3 expression could be associated with the damage of keratinocytes triggered by inflammatory mediators. Indeed, it has been reported that in LS, an initially hyperplasic epidermis is followed by a process of keratinocyte death, probably due to subepithelial lymphocyte infiltrates which cause apoptosis of undifferentiated keratinocytes. Furthermore, IFN-γ, produced by activated T cells of epithelial infiltrates, is involved in the activation of CD8^+^ T lymphocytes which may then trigger keratinocyte apoptosis [31]. Also, TG2 could be involved in keratinocyte apoptosis, as demonstrated in several experimental models [57,58,59]. Even in lichen planus, a marked TG2 expression in keratinocytes undergoing apoptosis has been shown, supporting the hypothesis of TG2 involvement in the cascade of events leading to the apoptotic phenotype [60].

Keratinocytes are essential in maintaining the integrity of the epithelial basement membrane, but in turn keratinocytes require a signal from the basement membrane to prevent their apoptosis. Normal keratinocytes express several adhesion molecules needed to maintain cell-to-cell and cell-to-matrix contact [61,62]. The E-cadherin is a transmembrane adhesion protein belonging to the cadherin family, involved in cell-to-cell adhesion in the stratified squamous epithelium [62] and is expressed in normal skin [63]. It has been demonstrated that in immortalized cell lines, the E-cadherin maintains cell contacts, thus preventing apoptotic cell death. A previous study carried out in oral lichen planus demonstrated that the loss of E-cadherin compromises epithelial integrity and may be involved in the apoptosis of basal keratinocytes [63]. In our study, we also found a reduction in the expression levels of E-cadherin in specimens of foreskin with BXO in comparison to those without BXO. Interestingly, the E-cadherin as well as TG1 and TG3 expression was down-regulated in BXO patients. Therefore, we can assume that these proteins are involved in the loss of structural integrity of the foreskin affected by BXO.

The etiology of BXO is certainly complex and probably multifactorial. In light of our results, we can hypothesize that possible triggers (microtrauma, infectious causes, autoimmune conditions) may lead to an inflammatory response of the foreskin with damage of keratinocytes and loss of functional integrity of the epithelial barrier; this condition may result in increased susceptibility to triggers, leading to the maintenance and progression of the BXO in children.

Most of the studies investigating the molecular mechanisms of the pathogenesis of BXO were performed on adult populations. To our knowledge, this is the first study that focuses on the molecular pathways associated with BXO damage in a pediatric population, investigating the role of TG1, TG2, TG3, IFN-γ and E-cadherin.

Our data showed the overexpression of TG2 and IFN-γ in the foreskin from patients with BXO, suggesting a pivotal role in triggering and maintaining the inflammatory response to an autoimmune mechanism or to an infectious cause or to a chronic irritation. Furthermore, the decrease in the expression of TG1, TG3 and E-cadherin suggests their involvement in the maintenance of the structural integrity of the foreskin. However, further studies are needed to better clarify the involvement of TGs in molecular mechanisms underlying LS and BXO and as possible targets for therapy.

## 4. Material and Methods

### 4.1. Patient Recruitment

Fifty-five children undergoing circumcision for clinical diagnosis of primary or secondary (suspected balanitis xerotica obliterans) phimosis were enrolled. The mean age at diagnosis was 9.7 ± 2.3 years, ranging from five to 15 years. Fifteen boys with a histological diagnosis of BXO were included in this study. BXO was defined istologically as an epithelial-stromal lesion characterized by squamous atrophy or hyperplasia, band like infiltration, hyalinization of the papillar dermis, hyperkeratosis, pigment incontinence and/or dermal edema. Fifteen age-matched patients with clinical diagnosis of primary phimosis without histological diagnosis of BXO were used as control group. We excluded from the study nine patients with pre-operative treatment with corticoid ointment or cream and 16 patients with uncertain histological diagnosis (aspecific inflammation). Informed consent was obtained by both study groups. Foreskin samples from patients with and without BXO were examined for the analysis by quantitative real-time PCR of transcript levels of TG1, TG2, TG3, IFN-γ and E-cadherin.

### 4.2. Gene Expression Analysis

After sampling, foreskin tissues were immersed in 500 µL of RNA stabilization reagent (RNAlater; Life Technologies, Milan, Italy), and stored at −80 °C until RNA was isolated. Total RNA was isolated using TRIzol reagent (Life Technologies), and two micrograms of total RNA were reverse-transcribed with High-Capacity cDNA Archive kit (Life Technologies). Then, TG1, TG2, TG3 as well as IFN-γ and E-cadherin mRNA levels were analyzed by Sybr Green Real-Time PCR using β-actin as endogenous control.

Quantitative PCR reactions were set up in duplicate in a 96-well plate and were carried out in 10 µL reaction volume containing 1× Sybr Green Mastermix (Life Technologies), 0.1 µM specific primers and 25 ng RNA converted into cDNA. Real-time PCR was performed in a 7900HT Fast RealTime PCR System under standard thermocycling profile.

Data were analyzed using the 2^−ΔΔ*C*t^ relative quantification method, and values are presented as fold change relative to the group without BXO.

The primer sequences used were the following: 5′-GATTGTCTTCAAGAACCCCCTTCCC-3′ (sense) and 5′-TCATCTGACTCCAGTCCCATTGCTC-3′ (antisense) for TG1; 5′-CCTTACGGAGTCCAACCTCA-3′ (sense) and 5′-CCGTCTTCTGCTCCTCAGTC-3′ (antisense) for TG2; 5′-AGCCTGTGAACGTGCAGATGCTCTTC-3′ (sense) and 5′-TGATTGCAGGGAACTTGTTGCAGG-3′ (antisense) for TG3; 5′-GCAGCCAACCTAAGCAAGAT-3′ (sense) and 5′-TCACCTGACACATTCAAGTTCTG-3′ (antisense) for IFN-γ; 5′-TGAGTGTCCCCCGGTATCTTC-3′ (sense) and 5′-CAGTATCAGCCGCTTTCAGATTTT-3′ (antisense) for E-cadherin; 5′-TTGTTACAGGAAGTCCCTTGCC-3′ (sense) and 5′-ATGCTATATCACCTCCCCTGTGTG-3′ (antisense) for β-actin.

### 4.3. Western Blotting

For Western blot analysis, foreskin tissues were homogenized with RIPA buffer on ice. Then, 30 µg of proteins were loaded on a 10% denaturing SDS-polyacrylamide gel and transferred to nitrocellulose membranes. Blots were blocked with 5% non-fat dry milk at a room temperature for one hour and then incubated with mouse antibody against E-cadherin and β-actin (respectively, diluted 1:1000 and 1:5000 in TBS-T) at 4 °C overnight. After washing, blots were incubated with horseradish peroxidase-conjugated anti-mouse (diluted 1:3000 for E-cadherin and 1:15,000 for β-actin) for 2 h at room temperature. Immunoblots were developed with ECL Plus chemiluminescent detection system kit using Kodak film (GE Healthcare, Milan, Italy). Bands were scanned and quantified by densitometric analysis with ImageJ 1.45 software (NIH, Bethesda, MD, USA) (http://rsb.info.nih.gov/ij/) using β-actin for normalisation.

### 4.4. Statistical Analysis

All values are expressed as mean ± standard error (SE). Statistical analysis of gene and protein expression data was carried out using Student’s *t*-test for comparison between two groups, with *p*-values <0.05 considered significant. Pearson’s correlation analysis was used to describe the relationship between TG2 and IFN-γ mRNA levels.
